# Analysis of the microbial diversity in takin (*Budorcas taxicolor*) feces

**DOI:** 10.3389/fmicb.2023.1303085

**Published:** 2023-12-22

**Authors:** Xiaoping Ma, Weichen Wang, Lijun Cai, Mei Xiao, Fang He, Zhen Liu, Dong Chen, Ya Wang, Limin Shen, Yu Gu

**Affiliations:** ^1^Key Laboratory of Animal Disease and Human Health of Sichuan Province, College of Veterinary Medicine, Sichuan Agricultural University, Chengdu, China; ^2^Management Office of Tangjiahe National Nature Reserve, Qingchuan, China; ^3^Sichuan Provincial Center for Animal Disease Prevention and Control, Chengdu, China; ^4^College of Life Sciences, Sichuan Agricultural University, Chengdu, China

**Keywords:** takin, *Budorcas taxicolor*, gut microbiota, microbial diversity, 16S rDNA high-throughput sequencing, metagenomics, conditioned pathogen

## Abstract

**Introduction:**

The intestinal tract of animals is a complex and dynamic microecosystem that is inextricably linked to the health of the host organism. Takin (*Budorcas taxicolor*) is a threatened species, and its gut microbiome is poorly understood. Therefore, this study aimed to analyze the microbial community structure and potential pathogens of takin.

**Methods:**

Takin fecal samples were collected from five sites in a nature reserve to ensure the uniformity of sample collection, determine the effects of different geographical locations on gut microbes, and analyze the differences in microbial communities between sites. Subsequently, high-throughput 16S rDNA gene sequencing was performed to analyze the microbial diversity and potential pathogens in the gut; the findings were verified by isolating and culturing bacteria and metagenomic sequencing.

**Results and discussion:**

The takin gut microflora consisted mainly of four phyla: Firmicutes (69.72%), Bacteroidota (13.55%), Proteobacteria (9.02%), and Verrucomicrobiota (3.77%), representing 96.07% of all microorganisms. The main genera were *UCG-005* (20.25%), *UCG-010_unclassified* (12.35%), *Firmicus_unclassified* (4.03%), and Rumino *coccsea_unclassified* (3.49%), while the main species were assigned to *Bacteria_unclassified*. Potential pathogens were also detected, which could be used as a reference for the protection of takin. *Pseudomonas* presented the highest abundance at Shuichiping and may represent the main pathogen responsible for the death of takin at the site. This study provides an important reference for investigating the composition of the bacterial community in the intestine of takin.

## 1 Introduction

Takin (*Budorcas taxicolor*) is a large herbivore in the family of Bovidae. Recent mitochondrial studies have shown that takin is more closely related to various sheep and that the similarity of takin and muskoxen is probably due to convergent evolution (Zhu et al., 2018). It is distributed mainly in the dense forests of the eastern foothills of the Himalayas, in Southwest and Northwest China, as well as in Nepal, Bhutan, India, and Myanmar, and migrates with the seasons (Zhuang et al., 2022). Human activities such as hunting and deforestation and the consequent loss and fragmentation of habitats have led to a decline in the takin population (Li et al., 2021). Takin is listed as Vulnerable in the IUCN Red List of Threatened Species.

The animal intestine is a complex and dynamic microecosystem in which microorganisms, including bacteria, viruses, fungi, and some small protozoa, are abundant (Yang et al., 2022). These microorganisms live in the intestinal tract and help the host fulfill various physiological and biochemical functions, which play a key role in animal health (Bou Zerdan et al., 2022). In the past decade, the development and improvement of next-generation sequencing and other omics technologies have enabled to study host-associated microbial communities in mammals, especially ruminants (Mtshali et al., 2022).

The Tangjiahe National Nature Reserve in Sichuan Province has a large takin (*Budorcas taxicolor tibetana*) population owing to increased protection. The Sichuan takin in this reserve has become increasingly common (Xiao et al., 2022). However, a small number of individuals die each year. In 2012, scientists conducted a detailed study on the status quo of takin and inspected the surrounding communities of the Tangjiahe Reserve. However, they were unable to reach a definite conclusion as to the cause of mortality (Cui, 2012).

The purpose of this study was to analyze the structure and potential pathogens of the intestinal bacterial community of takin sampled from five major protection stations in the Tangjiahe region. Metagenomics was used to verify the results. The findings provide insights into the core intestinal flora of takin for further study of its potential pathogens.

## 2 Materials and methods

### 2.1 Materials and methods for 16S rDNA sequencing

#### 2.1.1 Sampling

Samples were collected from the Tangjiahe National Nature Reserve in China (32° 59′ N, 104° 77′ E). Eighty-two takin feces samples were collected in November 2021 and allocated to five categories according to the conservation station. The fecal samples collected in Baiguoping, Motianling, Caijiaba, Baixiongping, and Shuichiping were named GI, GII, GIII, GIV, and GV, respectively. The fecal samples were stored in aseptic centrifuges, and each sample was collected with a separate tool. Samples collected on site were immediately placed in −20°C portable refrigerators and subsequently loaded with liquid nitrogen for further processing in the laboratory. A sample map is shown in Supplementary Figure S1.

#### 2.1.2 Isolation and identification of bacteria

The feces were inoculated into brain heart immersion (BHI) broth at 37°C for 12 h for constant temperature oscillation culture, and then the fecal broth mixture was inoculated into MacConkey agar and blood agar, cultured in the incubator at 37°C, and monitored every 12 h. A single colony was obtained by isolating and purifying colonies that presented different shapes and colors. The V3–V4 region was amplified using universal primers 341F (5′-CCTACGGGNGGCWGCAG-3′) and 805R (5′-GACTACHVGGGTATCTAATCC-3′). The PCR mixture was made up of 2.0 μL of DNA template, 12.5 μL of 2 × Taq PCR super-pre-mix, 1.0 μL of forward and reverse primers and 8.5 μL of ddH_2_O. The cyclic conditions were as follows: 30 s of initial denaturation at 98°C; 30 s at 98°C, 30 s at 55°C, 1 min at 72°C; and 2 min at 72°C. PCR products were sent to Healthy Creatures (Chengdu, China) for sequencing, and the data were analyzed by NCBI BLAST. The data were then compared to the NCBI’s GenBank sequence data available, and a tree was built using MEGA5 to compare the sequencing data.

#### 2.1.3 DNA extraction

DNA was extracted from different samples using cetyltrimethyl ammonium bromide according to the manufacturer’s instructions (Sigma-Aldrich, St. Louis, MI, USA). The reagent, designed to detect DNA from trace samples, is effective in extracting DNA from most bacteria (Ning et al., 2022). Nuclear-free water was used as the empty control. Total DNA was eluted at 80°C in 50 μL of an elution buffer and sent to LC-Bio Technology Ltd. (Hangzhou City, Zhejiang Province, China) for PCR measurement.

#### 2.1.4 PCR amplification and 16S rDNA sequencing

We amplified the V3–V4 high variable region of the 16S rDNA gene sequence using specific primers (16S V3–V4: 341F: 5′-CCTACGGGNGGCWGCAG-3′, 805R: 5′-GACTACHVGGGTATCTAATCC-3′) (Logue et al., 2016). The 5′ end of the primer was labeled with a specific barcode and a universal primer for each sample. The PCR amplification was performed in a 25-μL reaction mixture. The mixture contained 25 ng of template DNA, 12.5 μL of PCR pre-mix, and 2.5 mL of PCR-grade water per primer (Zhang et al., 2020). PCR conditions for amplifying the prokaryotic 16S fragment included 30 s of initial degeneration at 98°C, 10 s of degeneration at 98°C for 32 cycles, annealing at 54°C for 30 s, extension at 72°C for 45 s, and final extension at 72°C for 10 min (Kyei-Baafour et al., 2020). PCR products were identified with 2% agarose gel electrophoresis. To eliminate the possibility of false positive PCR, ultrapure water was used instead of sample solution in the whole DNA extraction process. Purification of the PCR products using AMPure XT beads (Beckman Coulter genomics, Danvers, Massachusetts, USA) was performed with Qubit (Invitrogen, Waltham, Massachusetts, USA). Amplified sub-pools were prepared for sequencing, and the size and number of amplified sub-pools were assessed using the Agilent 2,100 Biological Analyzer (Santa Clara, California) and Illumina Library Quantitative Kapa Biosciences (Wobby, Massachusetts, USA), respectively. The library was sequenced on the NovaSeq PE250 platform (Illumina, San Diego, CA, USA).

#### 2.1.5 Data analysis

Samples were sequenced on the Illumina NovaSeq platform according to the manufacturer’s recommendation (LC-Bio Technologies, Hangzhou, China). According to the unique bar code of the sample, the paired-end data was allocated to each sample, and the bar code and the primer sequence were removed. Flash merge was used for paired reads. We used fqtrim (v0.94) to filter raw readings under specific filtering conditions for high quality cleaning of labels. The Vsearch software (v2.3.4) was used to filter chimeric sequences (Zhou et al., 2021). Then, DADA2 was used to extract the characteristic table and sequence. Alpha and beta diversity were calculated by random normalization of the same sequence. The relative abundance of each sample was then normalized according to a SILVA (version 138) classifier (Devika et al., 2023). Alpha diversity was used to analyze the complexity of sample species diversity through five indices, including the Goods_coverage, Chao1, observed species, Simpson, and Shannon indices. QIIME2 was used to calculate the alpha diversity index and beta diversity in the samples. Graphs and diagrams were drawn using the R package (v3.5.2) (Xie et al., 2020). Blast was used for sequence alignment, and the feature sequences were annotated with the SILVA database for each representative sequence.

### 2.2 Materials and methods for metagenomics

#### 2.2.1 DNA extraction

As instructed by the manufacturer, the CTAB method was used to extract DNA from different samples. The reagent aims to extract DNA from trace samples and is effective in preparing DNA for most bacteria. The blank of the sample was composed of unused DNA swabs, which were examined for the absence of the DNA intensifier. According to the procedure described by the manufacturer (QIAGEN), total DNA was eluted in a 50-μL elution buffer, stored at −80°C, and sent to LC-BIOTECHNOLOGIES (Hang Zhou, Zhejiang Province, China) for PCR measurement.

#### 2.2.2 DNA library construction

DNA libraries were constructed using the TruSeq Nano DNA LT Library Preparation Kit (FC-121-4001). DNA was fragmented by dsDNA Fragmentase (NEB, M0348S) and incubated at 37°C for 30 min. The construction of a library started with a fragment of cDNA. A combination of filling reaction and exonuclease activity was used to produce blunt-end DNA fragments, and microspheres were purified for size selection. The A-base was then added to the blunt ends of each chain to prepare them for connection to the index adapter. Each adapter contained a T-base projection that attaches the adapter to the A-tail DNA. These junctions contained complete sequencing primer hybridization sites for single-ended, paired ends, and indexed reading segments. Ligation of a single or birefringent junction to a fragment was amplified by PCR at 95°C for 3 min; eight cycles at 98°C for 15 s, at 60°C for 15 s, at 72°C for 30 s, and then at 72°C for 5 min.

#### 2.2.3 Data analysis

We processed raw sequencing reads to obtain valid reads for further analysis. First, cutadapt (v1.4) was used to remove the sequencing adapters from the sequencing reads. Second, low-quality reads were trimmed using fqtrim (v0.94) using a sliding-window algorithm. Third, the reads were compared with the host genome using Bowtie 2 (v2.2.0) to remove host contamination. Once quality-filtered reads were obtained, they were reassembled to build the macro genome for each sample using MEGAHIT (v1.2.9). All coding regions (CDS) of metagenomic contigs were predicted by MetaGeneMark (v3.26). All of the CDS sequences were grouped with CD-HIT (v4.6.1) unigenes. Unigene abundance of a given sample was estimated using TPM. The number of alignment reads was determined using Bowtie2 (v2.2.0). The lowest common ancestor taxonomy of unigenes was obtained by aligning them against the NCBI NR database using DIAMOND (v 0.9.14). Similarly, the functional annotations (GO, KEGG, eggnog, CAZy, CARD, PHI, MGEs, VFDB) of unigenes were obtained. Based on the taxonomic and functional annotations and abundance profile of unigenes, differential analysis was performed at each taxonomic, functional, or gene-wise level using Fisher’s precise test (non-replicated groups) or the Kruskal–Wallis test (replicated groups).

## 3 Results

### 3.1 Bacterial isolation and identification

*Pseudomonas* was found to be the dominant bacterium in the feces, indicating that *Pseudomonas* was indeed present in the intestine of takin. In addition, we identified *Shigella*, *Escherichia coli*, *Escherichia ferguson*, and *Kronobacillus*. The evolutionary tree of the bacteria is shown in Supplementary Figure S2. NCBI accession numbers are shown in Supplementary Table S1.

### 3.2 16S rDNA gene V3–V4 region sequencing data characteristics

Amplicon sequence variants (ASVs) are identified based on high-throughput sequencing analyses of the DNA sequences in a microbial community, and the ASV ID number of each microorganism is ultimately obtained. ASV IDs represent the IDs of ASV feature sequences after QIIME2 noise reduction. The IDs were unordered and meaningless strings. The numbers represent the count of the feature sequence for each sample. The ASV IDs obtained in this study are listed in Supplementary Table S2.

A total of 82 samples of takin feces were collected from five protection stations in the Tangjiahe area. A total of 6,667,521 effective sequences were obtained by 16S rDNA amplicon sequencing after double-end splicing, quality control, and chimerism filtering. Of the sequences, 99.88% were between 400 and 500 bp in length, which was close to the primer length. The results are shown in Supplementary Figure S3.

### 3.3 ASV and dilution curve analysis

**Figure 1:**
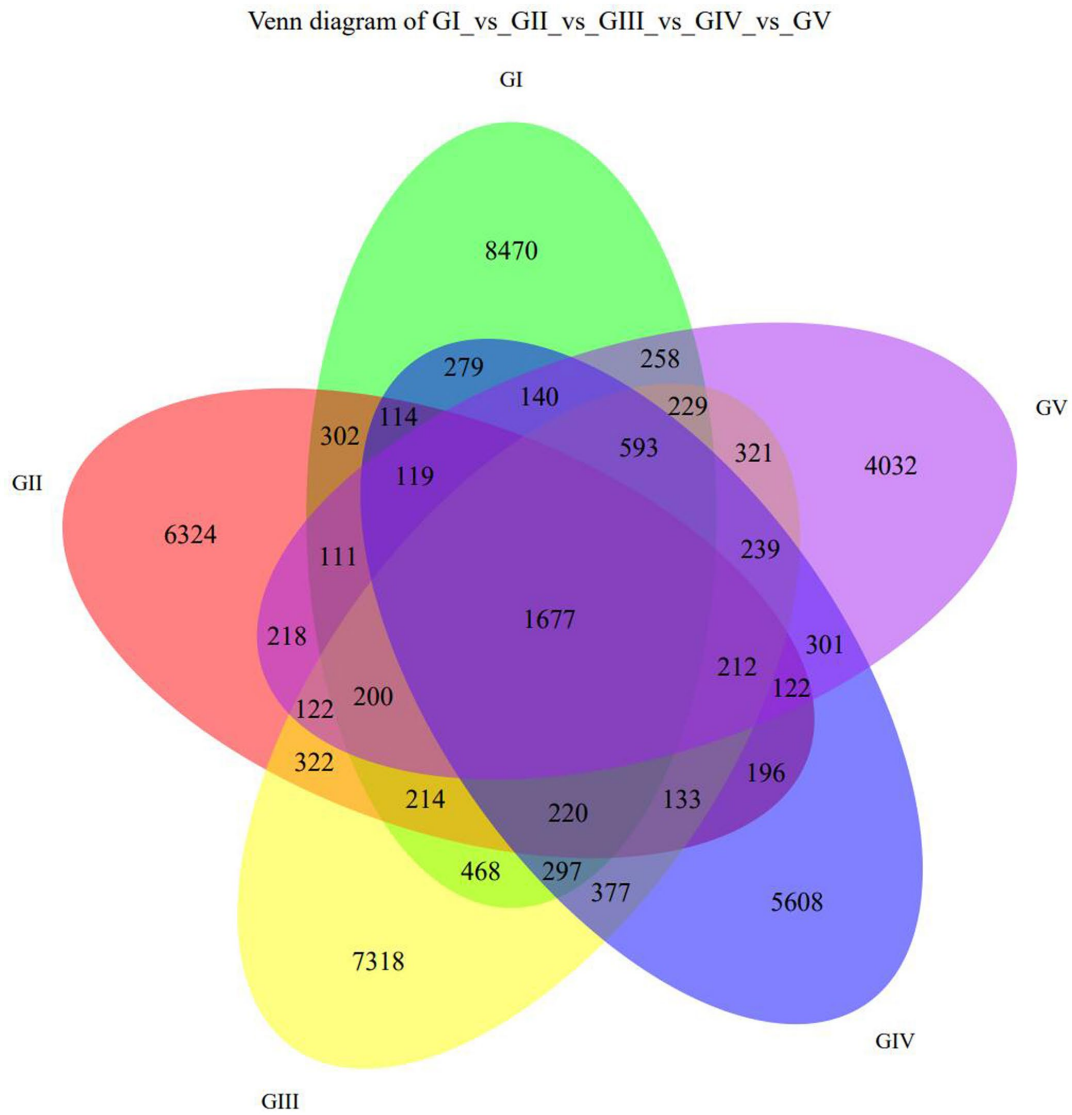
Venn diagram showing the ASV distribution at the five protection stations. GI, BaigguoPing; GII, MotianLing; GIII, Cai Jiaba; GIV, Bai Xiong Ping; GV, Shuichiping.

The Venn diagram (Figure 1) shows the five protection stations in the Tangjiahe area, including a large number of stool ASVs and the unique and shared ASVs between each protection station. BaigguoPing, MotianLing, Cai Jiaba, Bai Xiong Ping, and Shuichiping Protection Stations are represented by GI, GII, GIII, GIV, and GV, respectively. The five protection stations shared 1,677 ASVs, while GI presented 8,470 unique ASVs that were not shared with the other stations, thus making it the largest protection station (Figure 1).

**Figure 2:**
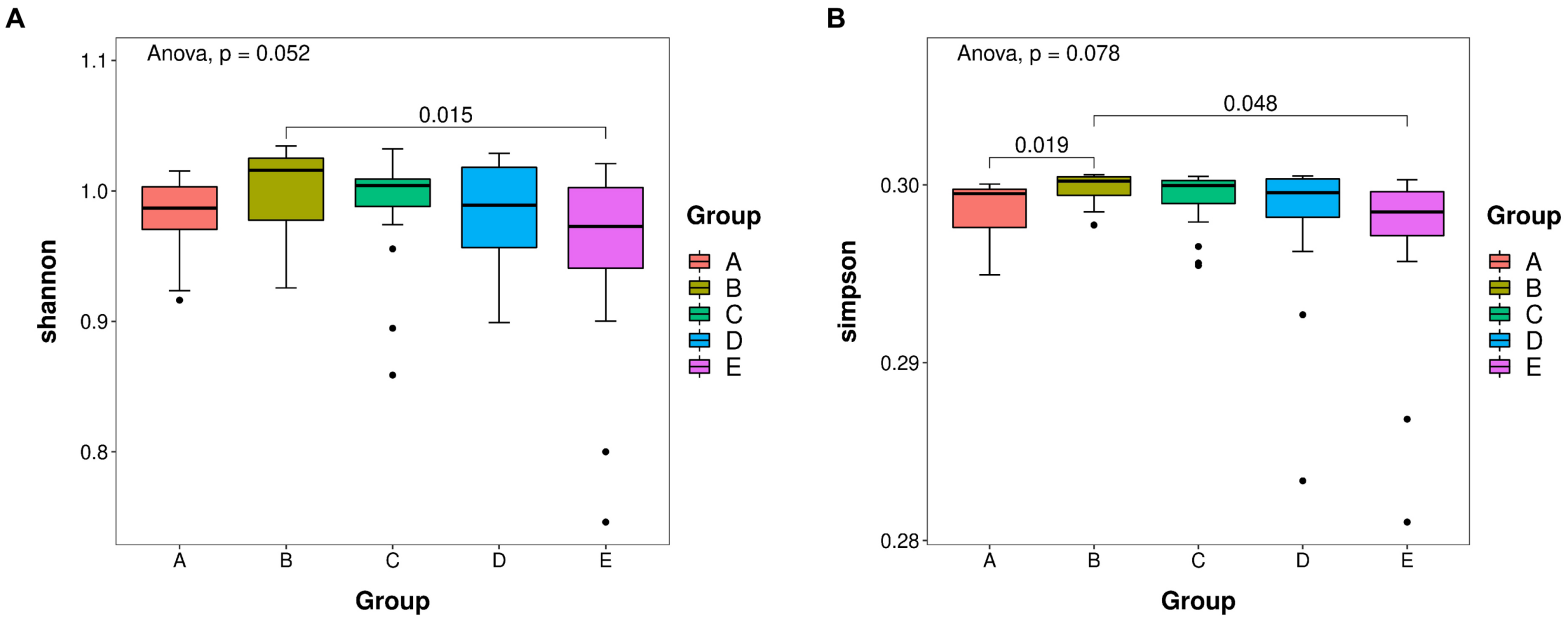
**(A)** Shannon boxplot. **(B)** Simpson boxplot. Species richness of takin intestinal bacteria was determined using 16S rDNA sequencing. The value of *p* indicates the parameter variance of all groups in the plot using the paired *t*-test. A–E represent the GI–GV regions, respectively.

The re-sampling procedure was simulated by the dilution curve, the variation tendency of the species was observed, and the species richness was estimated. The ASV richness was calculated by plotting the dilution curve, and the difference in microbial species diversity was explained by comparing the dilution curves of different samples. The Shannon index reflects species diversity in the community, and Goods_coverage indicates whether the sequencing results represent the actual sample (Figure 2). The abscissa represents the random number of sequences, and the ordinate represents the exponential size of each sample when the same number of sequences is extracted. We plotted all samples and groups together. Through this graph, the richness and diversity of each sample can be measured to some extent. From the dilution curve, we could also estimate the adequacy of the sequencing data as a reference indicator. A plateauing curve indicates that the sequencing data are representative, and that more data would only produce a small number of new species (Ma et al., 2022). Supplementary Figure S4 shows that the dilution curve of Goods_coverage tended to plateau within 2,000 sequences. The Shannon dilution curve plateaued in less than 2,000 sequences (Supplementary Figure S4B). These results indicate that the sequencing data were reasonable and credible and that additional data would only produce a small number of new species. The results of the remaining algorithms are shown in Supplementary Figure S5.

### 3.4 Comparison of bacterial diversity

Alpha and beta diversity analyses were performed to determine the bacterial diversity at the five protection stations and illustrate differences in the microbial community structure at each station. The Shannon index was the highest in GII and the lowest in GV (Figure 2A). The diversity of bacteria in GV significantly differed from that in GI (Figure 2B). The bacterial species diversity in GII was higher than that in the samples from the other four protection stations.

**Figure 3:**
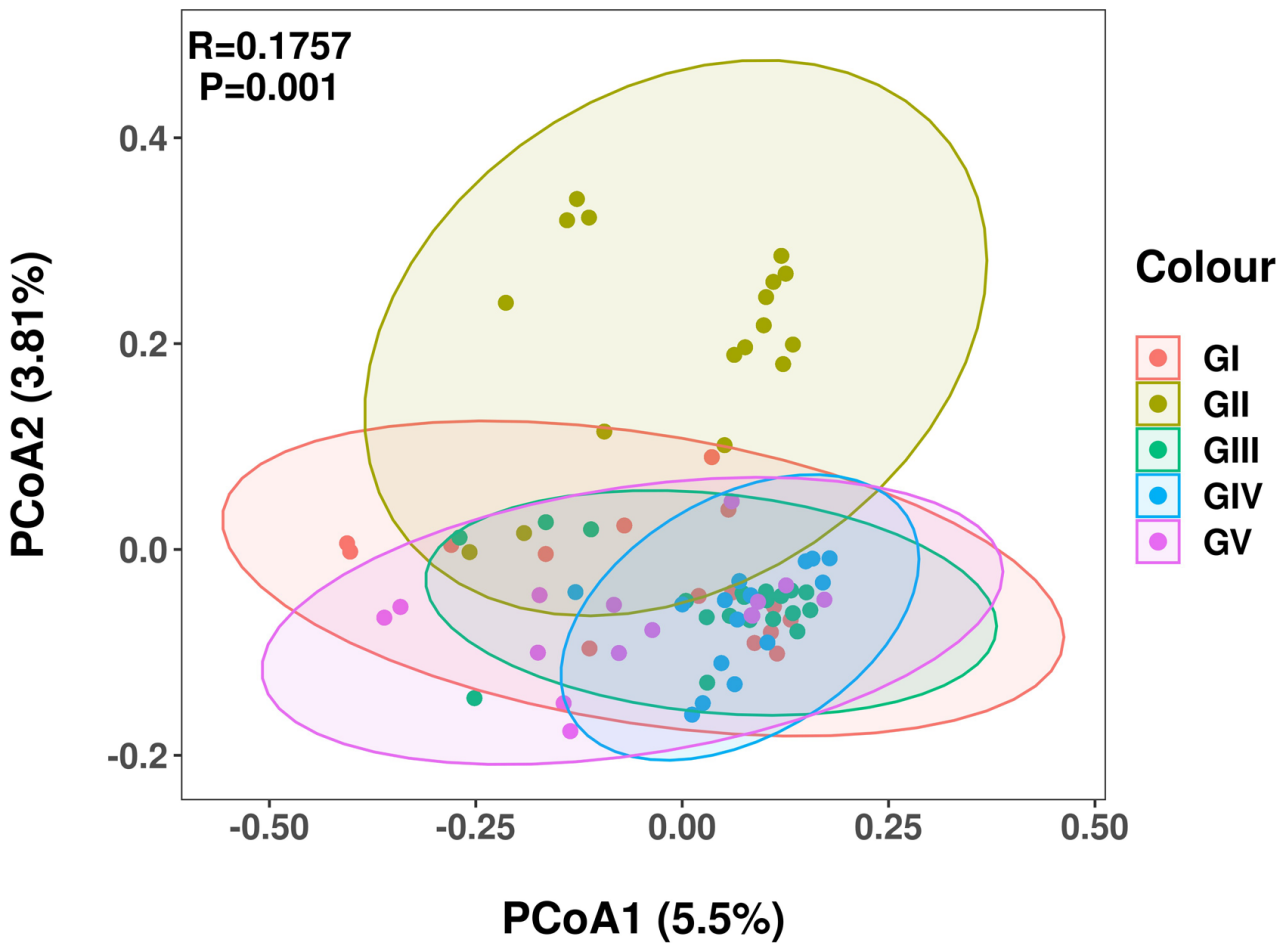
Principal coordinate analysis of intestinal microbial communities of takin. Different colors represent different protection areas; the closer the sample, the more similar the microbial composition.

Principal coordinate analysis (PCoA) analysis was used to compare the fecal flora of the takin specimens from the five protection stations. The different colors in the results represent different groups, and the closer the samples are, the more similar the microbial composition and structure between the samples, the less differentiated. The percentage of the horizontal and ordinate indicates how well the first and second axes explain the sample differences. Except for GII, the fecal flora of GIV was more concentrated than those of the other protection stations, with no obvious difference between them (Figure 3).

### 3.5 Analysis of differences in composition and abundance of bacteria

**Figure 4:**
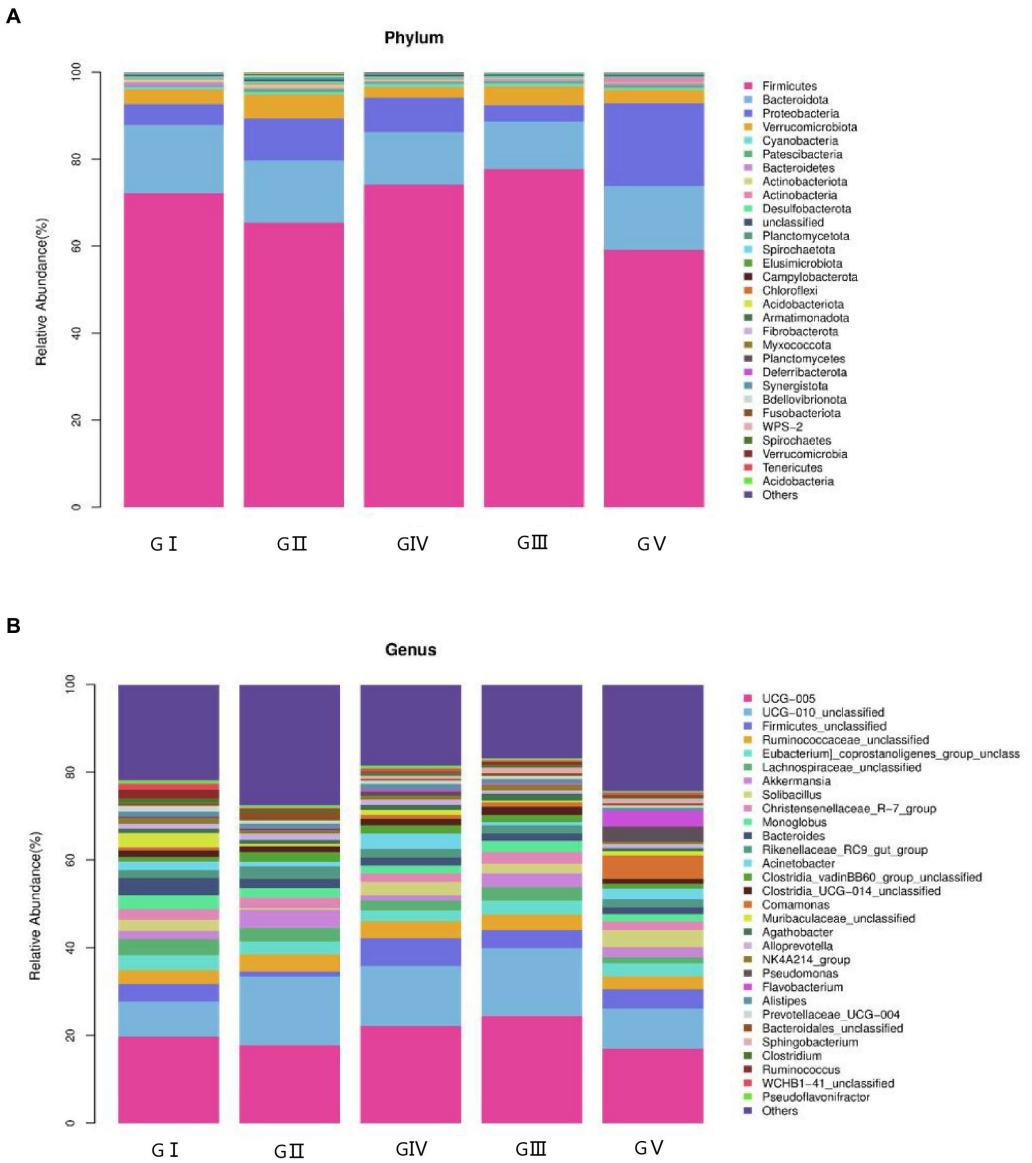
Stacked bar charts showing the percentage of takin gut bacteria at the phylum **(A)** and genus **(B)** levels.

The coverage rate of the sequencing exceeded 97% based on the results of ASV annotation. In total, 48 bacterial families, 223 orders, 390 families, 953 genera, and 1,653 species were obtained. To determine the differences in bacterial composition and abundance, a bar chart was used to illustrate the differences in colony distribution and abundance at the phylum and genus levels. At the phylum level, Firmicutes was the most abundant, except for GV, with a relative abundance of over 60%, followed by Bacteroides, Proteobacteria, and Verrucobacteria. The total abundance of these four phyla accounted for more than 95% of all phyla, indicating the absolute dominance of these phyla (Figure 4A). According to the macrogenomic sequencing results, Firmicutes, Bacteroides, and Proteobacteria were the dominant phyla, which was in line with the results of the 16S rDNA high-throughput sequencing (Supplementary Figure S6).

**Figure 5:**
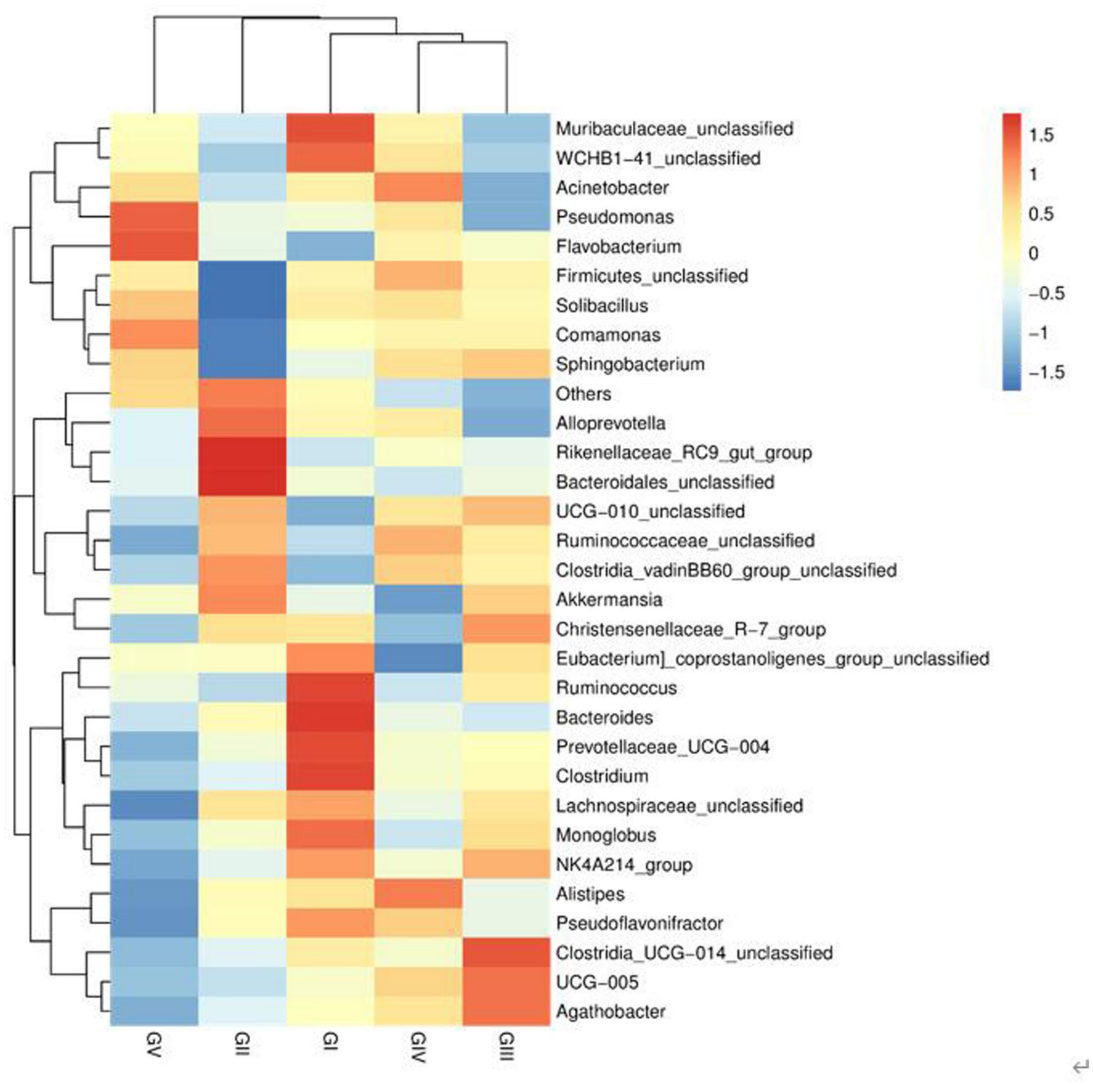
Heatmap of bacteria. Each row represents a species, and each column represents a sample/group. The blue-to-red gradient in the graph reflects the change in abundance from low to high. The closer to blue, the lower the abundance; and the closer to red, the higher the abundance.

At the genus level, the four most abundant bacterial genera were *UCG-005, UCG-010_unclassified, Firmicides_unclassified*, and *Rumino coccsea_unclassified* (Figure 4B). The community composition data of the top-30 genera were clustered according to the abundance distribution or the similarity between samples, and the taxa and samples were sorted according to the clustering results. High and low abundances of taxa can be distinguished by clustering, and the similarity and difference of the components of samples can be reflected by color gradients and similarity. The dominant bacterium at the five protection stations was *UCG-005*, which was the most abundant and positively correlated in GIII (Figure 5). The results for the 30 most abundant bacteria at the genus level are shown in Supplementary Table S3, which also indicates that the relative abundance of bacteria in GV and GIV was lower than in the samples from the other three sites, similar to the results shown in Figure 1.

**Figure 6:**
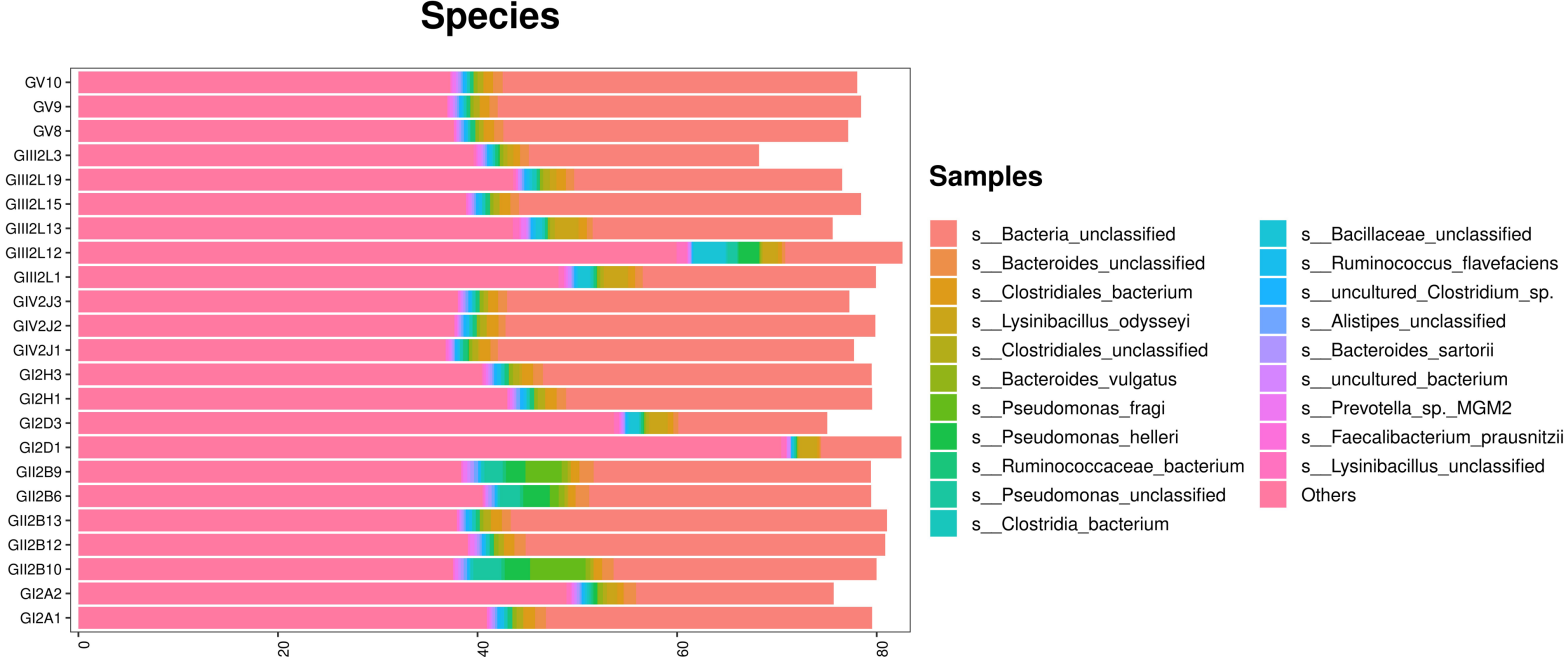
Metagenomic sequencing stacked bar charts showing the percentage of takin gut bacteria at the species levels.

The metagenomic sequencing results confirmed the above results. In addition, at the species level, the highest abundance was found for *Bacteria_unclassified*, which accounted for more than 40% of all bacterial species. The remaining species included *Bacteroids_unclassified*, *Clostridiales_bacterium*, *Lysinibacus_dysoseyi*, *Cycles_unclassified*, and *Bacteroids_vulgatus*. Similarly, *Pseudomonas* species, such as *Pseudomonas_fragi*, were also found via macro genome sequencing, which confirmed the bacterial isolation and culture results (Figure 6).

### 3.6 LEFSE analysis

**Figure 7:**
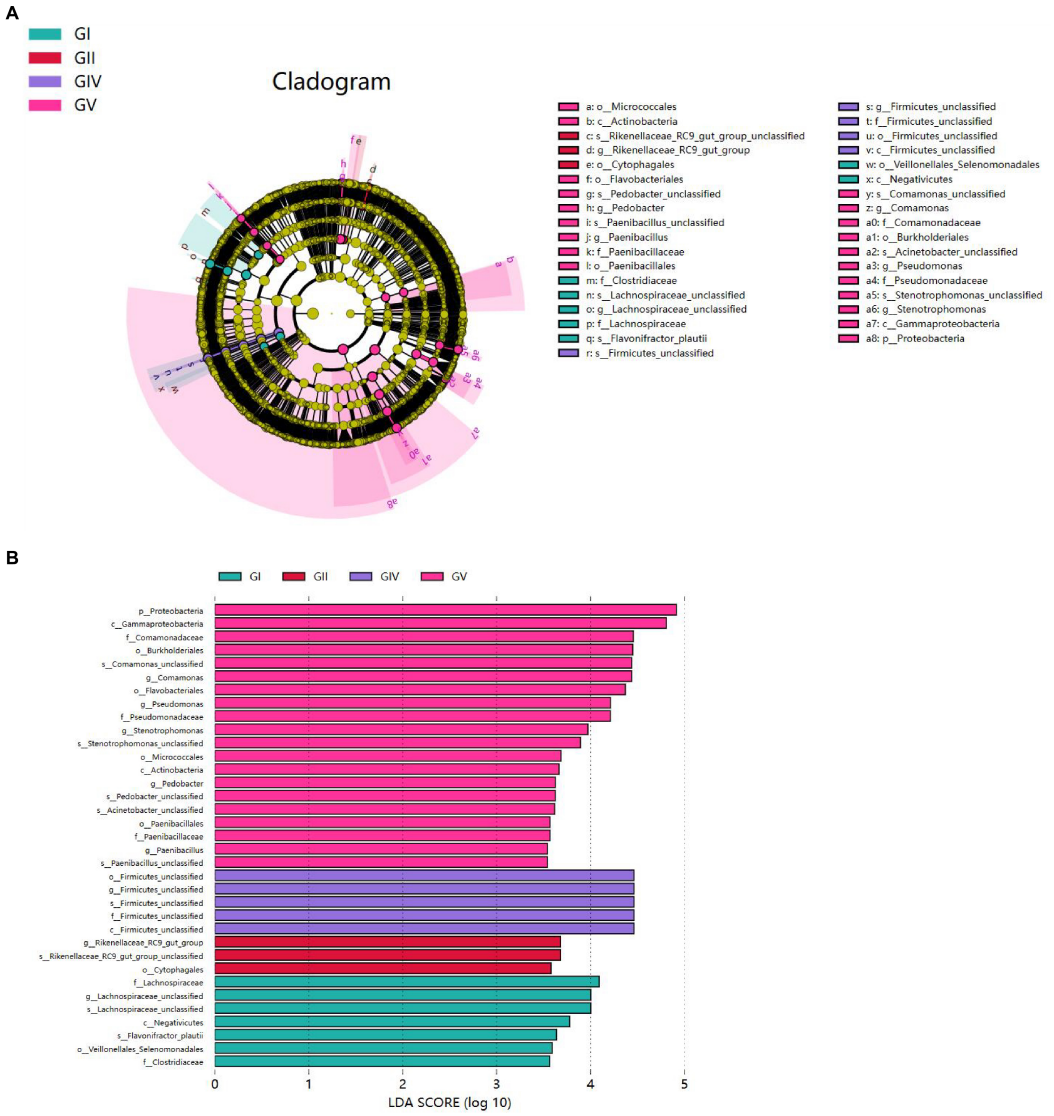
**(A)** Each node represents a species taxonomy level, and the more abundant the species, the greater the difference. **(B)** Bar chart showing the log LDA score for each species at the taxonomic level. The colors indicate the abundance of different species in the highest area; the length represents the influence of the LDA score and the significantly different species among different groups.

To analyze the differences in colony abundance among the five conservation stations, species with significant differences in abundance between different groups were identified. The evolutionary branching diagram of the Linear discriminant analysis Effect Size (LEfSe) analysis is shown in Figure 7A. The green, red, blue, and purple areas represent GI, GII, GIV, and GV, respectively. The yellow nodes indicate no significant differences among group, while red nodes indicate significant differences among groups, and the species had a high abundance in the red grouping. Significant differences are noted directly in the graph, and differences at other levels are represented by letters at the species node. The bar chart (Figure 7B) shows significantly different species with LDA scores greater than 3.5, that is, the statistically different biomarkers. The color of the bars indicates the abundance of species among different species, and the length represents the LDA score, that is, the impact of the significantly different species among different groups.

Proteobacteria presented significant differences in GV. At the genus level, significant differences were also found for *Comamonas*, *Pseudomonas*, *Stenotrophomonas*, *Pedobacter*, and *Paenibacillus* in GV; for *Firmicus_unclassified* in GIV; for *Rikenellsea_RC9_GUT_GROUP* in GII; and for *Achnospirsea_unclassified* in GI. These results suggest that these bacteria may be biomarkers for the corresponding areas.

### 3.7 Analysis of potential pathogens

**Figure 8:**
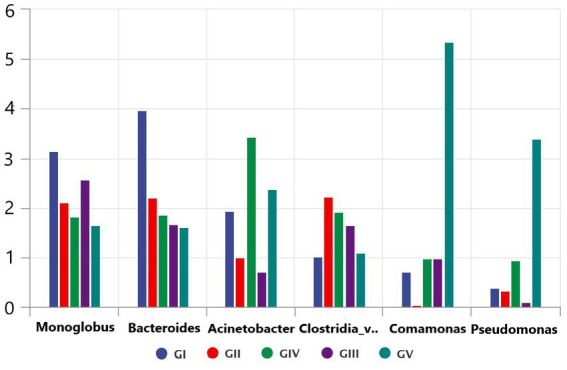
Comparative analysis of separate groups of pathogenic bacteria in the five protected areas.

Through the bacterial community and LEFSE analyses, we identified the potential pathogens, including *Monoglobus, Pseudomonas*, *Acinetobacter*, and *Clostridium*, which might play an important role in the death of takin in the Tangjiahe region because these pathogens can cause diseases in ruminants (Li et al., 2017; Bie et al., 2020; Wang et al., 2020). After analyzing the five bacterial groups, we found that *Salmonella* was the most abundant but less widely distributed in the Motianling Conservation Station, followed by *Monoglobus*, *Bacteroides*, and *Acinetobacter* (Figure 8). Among them, *Comamonas* was the most concentrated in GV and almost absent in GII. Similarly, the number of *Clostridia_vadinBB60_group_unclassified* pseudomonas in GV was significantly higher than in the samples from the other areas. Potential pathogens such as *Clastridialects_bacterium*, *Pseudomonas_fragi*, and *Pseudomonas_helleri* were also found in the macro genome sequencing species results.

### 3.8 Functional prediction

**Figure 9:**
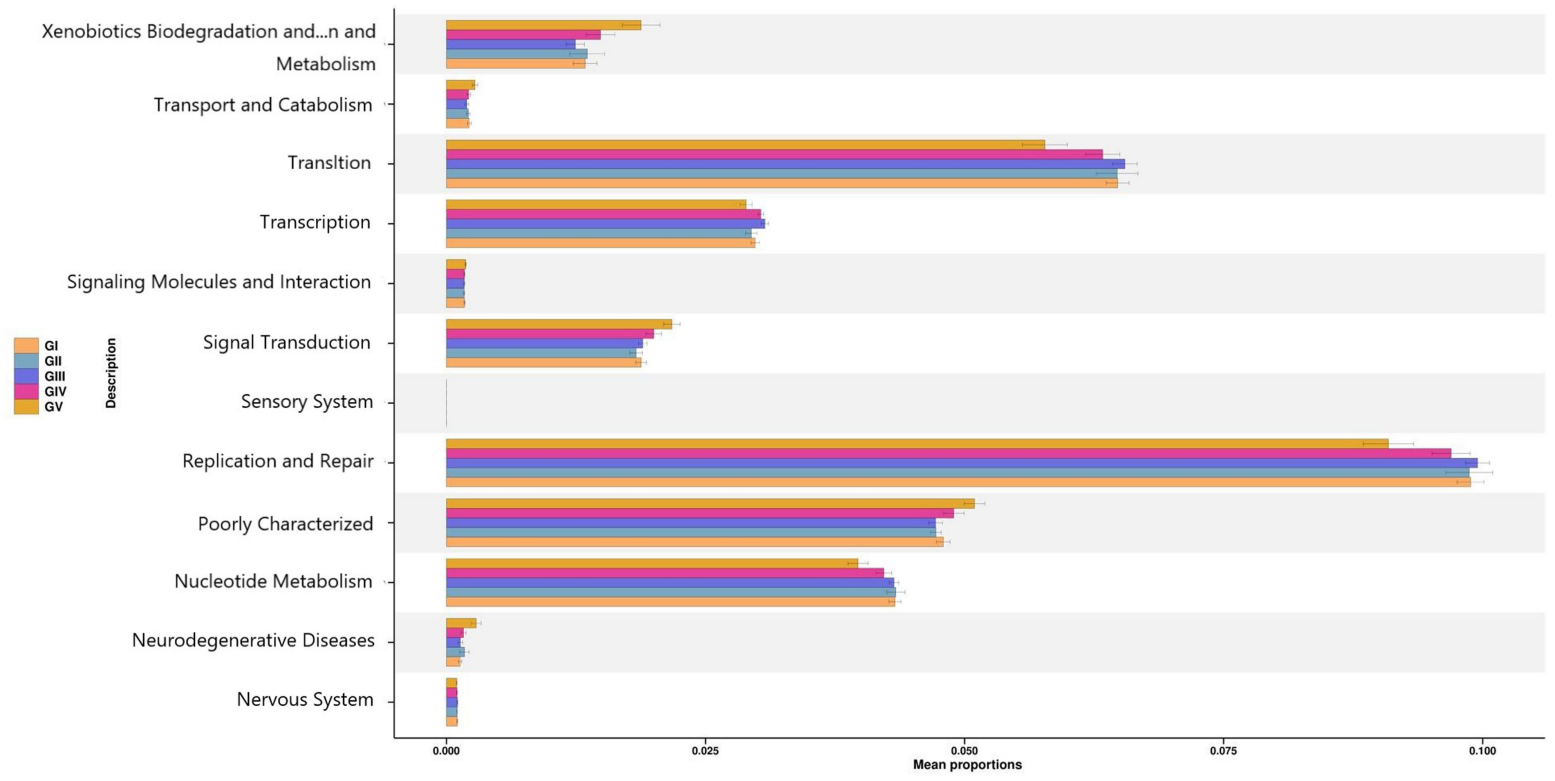
PICRUSt2 test result map of pathway differences (KEGG level 2). The horizontal axis represents the relative abundance of each group, and the vertical axis represents the function of statistically significant differences.

**Figure 10:**
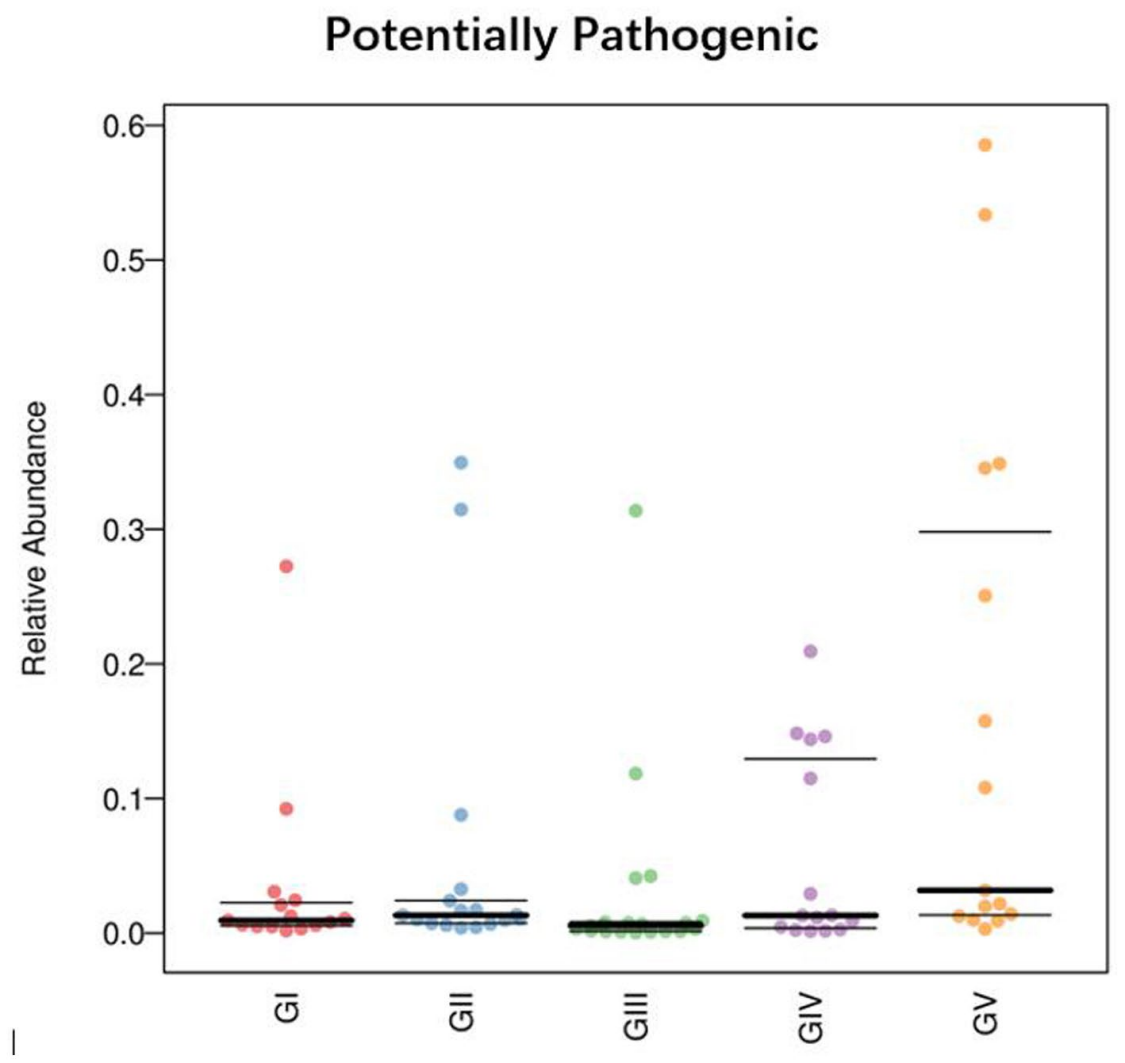
Comparison of bacterial phenotypes in samples. The scatter plot shows the relative abundance of each sample in different phenotypes in different groups.

To determine the influence of the intestinal microbiota on body functions, we performed a Kyoto Encyclopedia of Genes and Genomes (KEGG) pathway analysis to identify the gut microbe functions. PICRUSt2 was used to predict gene function in the five sample groups. At level 2, there was no significant difference among the five groups, mainly focusing on replication repair, translation, and nucleotide metabolism. However, genes associated with neurodegenerative diseases were also detected, suggesting that all five areas were infected with pathogens (Figure 9). Functional predictions for other levels are presented in Supplementary Figure S7. Predictions of bacterial phenotypes suggested that potential pathogens were the most abundant in the GV region (Figure 10; see Supplementary Figure S8 for further prediction of bacterial phenotype).

## 4 Discussion

Takin is an ancient animal with high ornamental and economic value (Zhu et al., 2018). However, environmental destruction caused by human activity has caused the takin population to decline. According to infrared testing results, there are currently approximately 1,300 Sichuan takins in Tangjiahe Nature Reserve, making their protection very important (Guan et al., 2015). However, persistent mortality of takins in the reserve has raised concern, and the cause of death has not been determined. In this study, the intestinal microflora of takin in the nature reserve was analyzed using 16S rDNA high-throughput sequencing.

Species diversity analysis using alpha diversity of fecal samples showed the Shannon and Simpson indices strongly differed between GII and GV. Differences in the Simpson index between GII and GI were also evident, and the same was true for GI and GIII. This showed that GII had the highest bacterial abundance and diversity. The PCoA analysis results revealed a significant difference in colony structure between GII and the other four groups, possibly due to geographical differences (Pandit et al., 2018). The different protection stations have different environments, including food, water, or soil. In addition, some protection stations included saltworks, which increase the salinity of the soil, and may thus also contribute to the observed difference (Li et al., 2016).

At the phylum level, the intestinal flora of takin in the Tangjiahe area was rich in Firmicutes, Bacteroidota, Proteobacteria, and Verrucomicrobiota. Similar results were obtained in the metagenomic sequencing. According to previous studies, the observed flora is structurally similar to that in the gut of other ruminants (Mtshali et al., 2022). Similarly, at the genus level, the structure of the gut microbiome, including core bacteria such as *UCG-005* and *UCG-010*, was similar to that of other ruminants (Wang et al., 2022). Most of these bacteria are the core flora of ruminants, which play an important role in promoting the maturation of the intestinal barrier (Neil et al., 2019). The acetate produced by Bacteroides can be absorbed by other bacteria, for example, *Butyricosus* and *Megasporus*. The butyrate and propionate produced by Bacteroides are the main energy sources for intestinal epithelial cells. Butyrate also inhibits signaling pathways of pro-inflammatory cytokines, boosting gut barrier function by increasing mucin secretion and enhancing tight connectivity (Chen et al., 2022). *UCG-005* belongs to the family Ruminococcaceae, which is the dominant microorganism in the ruminant rumen and plays an important role in the degradation of cellulose and polysaccharides (Zhi et al., 2022).

Through the KEGG pathway analysis, we found that most of the microorganisms were involved in metabolism. At level 2, these bacteria were involved in DNA replication, repair, and translation and nucleotide metabolism. However, a small number of microorganisms were associated with neurodegenerative diseases, which represent a group of neurological diseases in which neurons in the central or peripheral nervous system are progressively lost, including Alzheimer’s disease, bovine spongiform encephalopathy, and Creutzfeldt-Jakob disease. Such diseases can lead to impaired memory, cognition, behavior, sensation, and motor function in animals and humans (Dugger and Dickson, 2017). Studies have shown that ecologically disturbed peripheral immune responses can transmit bacterial and inflammatory signals throughout the body, including the brain, and that such immunomodulatory communication may lead to neurodegenerative diseases (Boeri et al., 2021). *Morganella* and *Citrobacter* were also observed in this study, and they have been shown to produce histidine decarboxylase (HDC), which induces histamine production and thus affects the immune system (Willmann et al., 2015).

We also found some potential pathogens, and although their relative abundance was not high, some of them may be associated with takin mortality. The most abundant pathogen was *Monoglobus*, a specialized bacterium that degrades pectin in the human colon (Kim et al., 2019). The high abundance of *Monoglobus* in the takin gut may be due to undigested feed in the large intestine (Sim et al., 2022). *Monoglobus*, a conditioned pathogen commonly found in human and animal intestines, can cause urinary tract, skin, soft tissue, and wound infections; lead to food poisoning or diarrhea; and cause pneumonia if it enters the lungs (Lin et al., 2013). Therefore, it has public health significance. Deaths from mixed infections of *Monoglobus* and *E. coli* were reported in 2019 (Bu, 2019). In addition, we detected other potential pathogens, such as *Bacteroides*, *Acinetobacter*, *Clostridia_vadinB60_group_unclassified*, *Comamonas. Clostridia_vadinB60_group_unclassified* belongs to the *Clostridia_vadinB60_group*, which is composed of conditional pathogens. The abundance of Acinetobacter increases significantly in winter, but its pathogenicity has not been discussed, and the abundance of this phylum in the current study was similar to that reported previously (Guan et al., 2022). Current research has found that the abundance of *Clostridia_vadinB60_group* positively correlated with the fasting insulin level and HOMA-IR, especially in overweight pregnant women or patients with preeclampsia (Yao et al., 2020). Although some conditional pathogens were found in takin feces, there is no evidence that these bacteria are responsible for the death of takin in protected areas, and their abundance does not reflect the health of the host. In the future, preventive and therapeutic measures must be taken to prevent the transmission of these pathogens between animals in protected areas or from animals to humans.

By analyzing potential pathogens, we found that they tended to be more contagious when an animal’s immune system was compromised. *Comamonas* was found in all five groups, with the highest abundance in GV and the lowest in GII, as was the case with *Pseudomonas*. At the species level, the abundance of *Pseudomonas_fragi* and *Pseudomonas_helleri* was also the highest in GII. This shows that in the reserve, takins carry conditional pathogens, perhaps due to environmental and other natural factors. Phenotypic predictions showed that the latent pathogenicity of the bacteria in Shuichiping (GV) was significantly higher than that in the other protection stations. *Pseudomonas* is a harmful bacteria in the intestinal tract. Studies have shown that cell surface signaling is related to virulence, biofilm formation, and cell–cell interactions (Llamas et al., 2014). Therefore, we believed that *Pseudomonas* is the potential pathogen with the greatest threat to takins in Shuichiping. The map of the Tangjiahe Reserve shows that Shuichiping is located in the center of the reserve and surrounded by rivers on all sides. Grazing activities are frequent, and the area is popular for tourism. Therefore, the chance that humans or animals carry conditional pathogenic bacteria is greater than that at the other conservation stations. Moreover, the takin immune systems is affected to varying degrees at the different stations, and the risk of infection may vary accordingly. However, according to our results, most of the natural flora in the intestine does not have adverse effects on takins, and only few pathogens were conditioned pathogens. This does not mean, however, that these pathogens are unrelated to takin death. When takins are compromised by other factors (such as parasites), bacteria exploit the opportunity to multiply and cause damage. Modulating and suppressing the immune system is a competitive advantage of many parasites, whereby they can influence the host’s immune response (Junginger et al., 2017). At the same time, in the host, the parasite is affected by additional antimicrobial agents produced by the host immune system and competing gut microbiota (Palmer-Young et al., 2016). In this case, parasite infection is likely to lead to host body immunity and imbalance of the intestinal flora so that the potential pathogen can gain access, such as *Salmonella*, *Campylobacter jejuni*, *Staphylococcus aureus*, etc. Therefore, this preliminary analysis of the adverse bacterial factors in takins provides insights for follow-up studies on pathogens and the protection of takins.

In addition, according to the results of our metagenomic sequencing, we also found a small number of common pathogenic bacteria, such as *E. coli* and *Salmonella,* at the genus level. Although we successfully isolated *E. coli* in bacterial isolation culture, we were unable to isolate it due to the extremely small abundance of *Salmonella*. Specific data are shown in Supplementary Table S4. *Escherichia coli* is a common pathogen in animal husbandry, which causes great harm to ruminants and diarrhea, septicemia, and mastitis (Matias et al., 2019). Most *E. coli* strains cause intestinal infections (Agarwal et al., 2022). For *Salmonella*, the general number of bacteria in the intestines of livestock and poultry is high when the resistance of animals is reduced due to disease, weakness, malnutrition, and fatigue. *Salmonella* in the intestine can enter the blood through mesenteric lymph nodes and tissue, causing systemic infection and even death (Phu Huong Lan et al., 2016). In our view, because of the low levels of these two bacteria, it is possible that the inherent flora in the gut of takin does not have harmful effects on animals when the flora is balanced.

Due to time and geography constraints, we could not entirely ensure that the collected samples were from dead takins. Future studies should consider both temporal and spatial factors and population samples, such as related to seasonal variations. For a more detailed analysis, sequencing should be focused on other types of microorganisms as well, such as fungi and parasites. Our study revealed the microbial structure of the takin gut, thereby providing a foundation for the protection of and future research on takins.

## 5 Conclusion

In this study, the intestinal microbial community structure of the rare animal takin was analyzed, and it was mainly composed of Firmicutes, Bacteroidetes, Proteobacteria, and Verrucobacteria. Analyses of the intestinal microbial community structure of takin from five conservation stations in the Tangjiahe Nature Reserve revealed differences among the stations. In particular, Motianling (GII) was significantly different from the other four protection stations and Baiguoping (GI) had the largest number of unique microflora. Due to the differences in geographical environment, each protection station apart from Caijiaba had corresponding biomarkers, such as *p_Proteobacteria* in Shuichiping, *g_Firmicutes_unclassified* in Baixiongping, *Rikenellaceae_RC_9_gut_group* in Motianling, and *g_Lachnospiraceae_unclassified* in Baiguoping. These bacteria regulated the metabolism of takin by participating in DNA replication, repair, and translation and nucleotide metabolism. Most of the bacteria were normal communities in the gut of ruminants, although we also found some potentially pathogenic bacteria. The virulence of *Pseudomonas* was affected by cell surface signaling and caused damage to the intestinal tract of animals, and it also represented the most important pathogenic microorganism underlying the death of takins in Shuichiping. *Morganella* and *Citrobacter* may cause neurodegenerative diseases in animals, and although their abundance was low, they are important pathogens of wildlife. The information presented in this study can be used as a reference for threatened animals.

## Data availability statement

The datasets presented in this study can be found in online repositories. The names of the repository/repositories and accession number(s) can be found in the article/Supplementary material.

## Ethics statement

The animal studies were approved by Sichuan Agricultural University Animal Ethics Committee and Sichuan Tangjiahe National Nature Reserve (permit number DYYS20211118). The studies were conducted in accordance with the local legislation and institutional requirements. Written informed consent was obtained from the owners for the participation of their animals in this study.

## Author contributions

XM: Funding acquisition, Resources, Supervision, Visualization, Writing – review & editing. WW: Data curation, Formal analysis, Methodology, Software, Validation, Writing – original draft, Writing – review & editing. LC: Resources, Validation, Visualization, Writing – review & editing. MX: Resources, Validation, Visualization, Writing – review & editing. FH: Resources, Validation, Visualization, Writing – review & editing. ZL: Data curation, Software, Validation, Writing – review & editing. DC: Resources, Validation, Visualization, Writing – review & editing. YW: Resources, Validation, Visualization, Writing – review & editing. LS: Resources, Validation, Visualization, Writing – review & editing. YG: Data curation, Supervision, Validation, Visualization, Writing – review & editing.
