# Supplementary figures and images for: Analysis of the microbial diversity in takin (Budorcas taxicolor) feces
Source: Front Microbiol. 2023 Dec 22;14:1303085. doi: 10.3389/fmicb.2023.1303085 (PMC10768053; doi:10.3389/fmicb.2023.1303085)

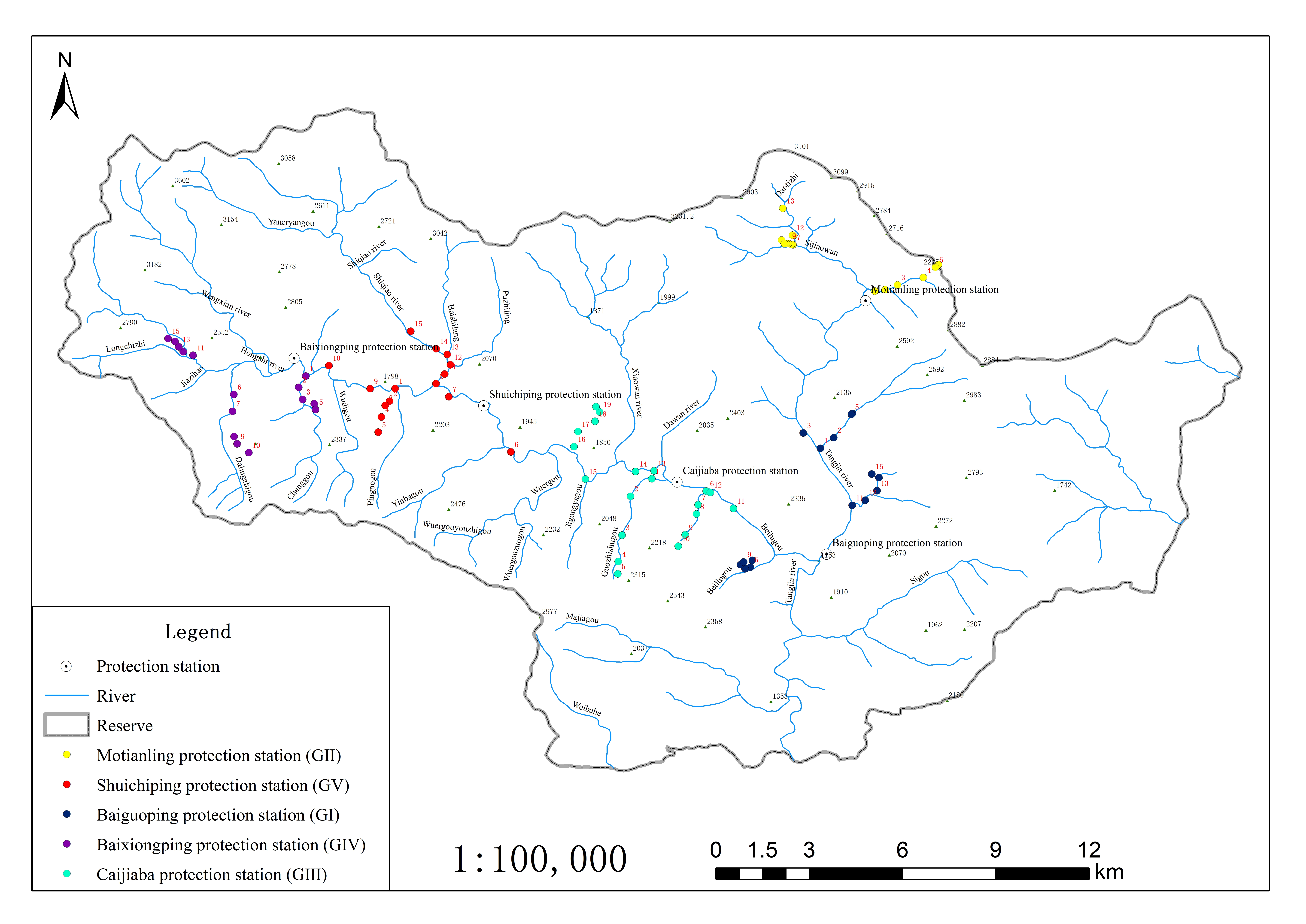

Supplement: Supplementary file 5 [file Image_1.JPEG]

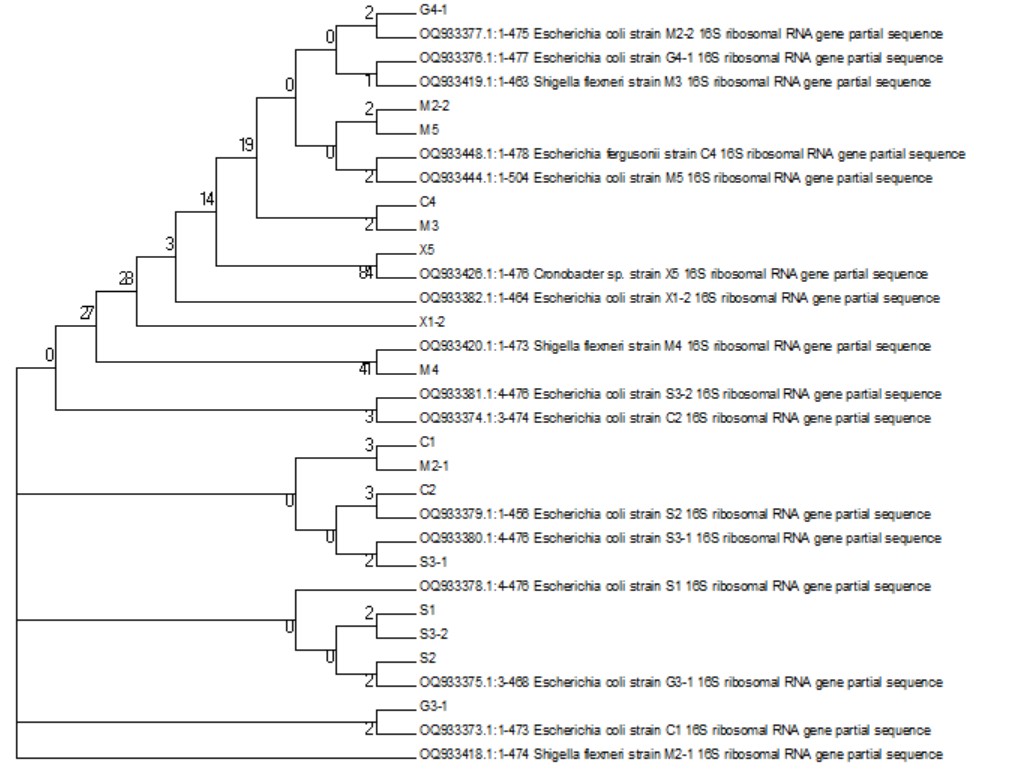

Supplement: Supplementary file 6 [file Image_2.JPEG]

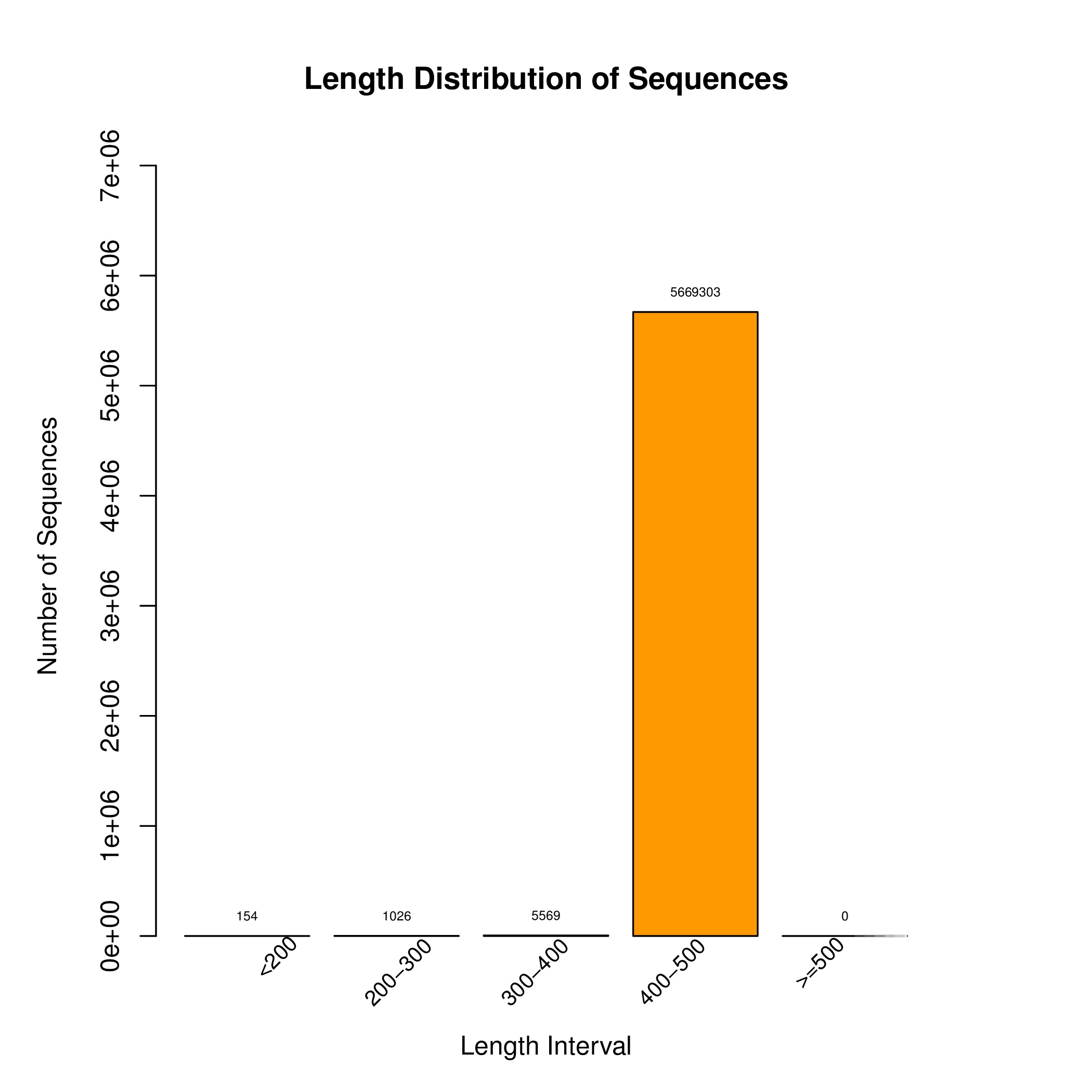

Supplement: Supplementary file 7 [file Image_3.JPEG]

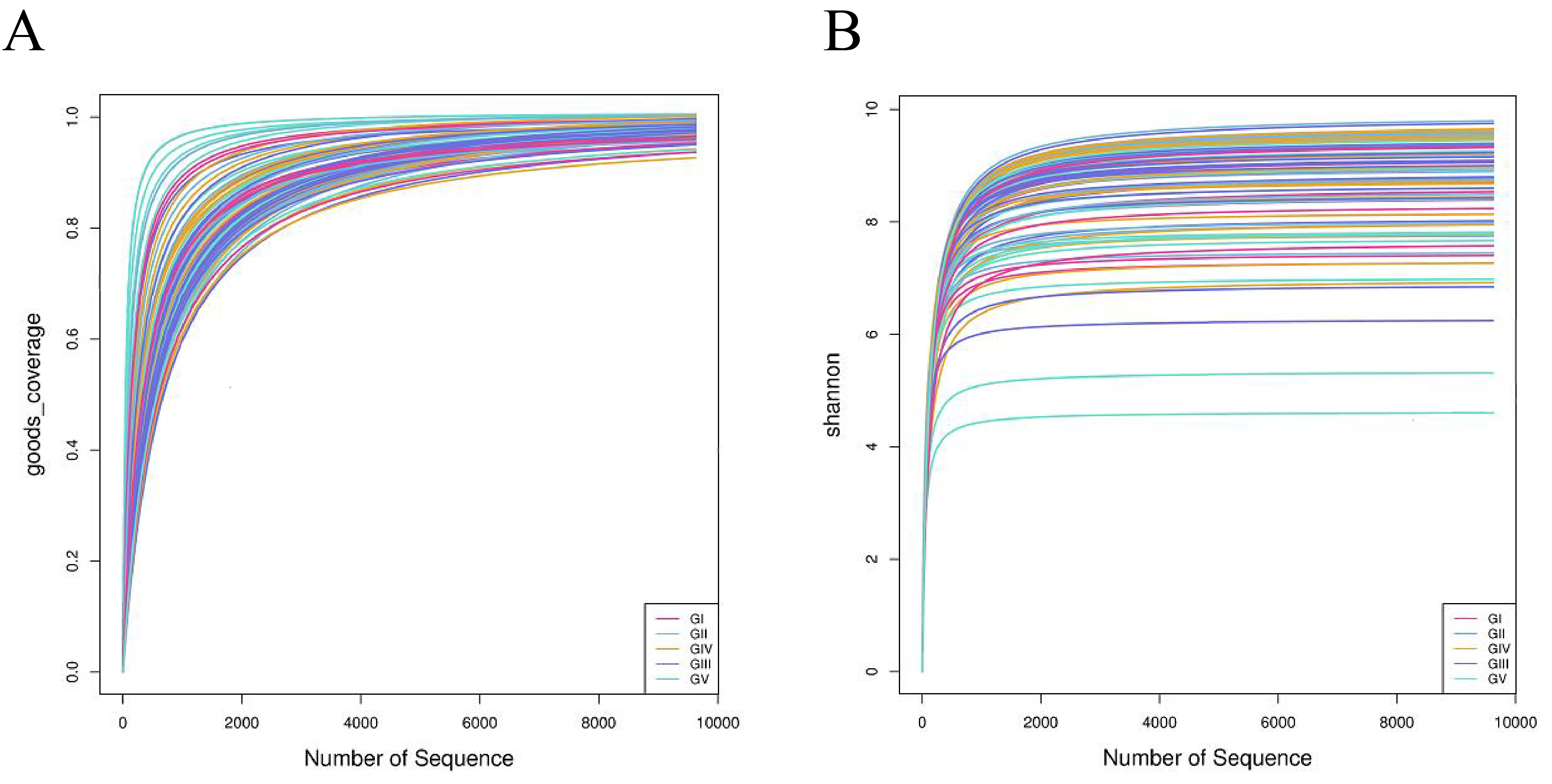

Supplement: Supplementary file 8 [file Image_4.JPEG]

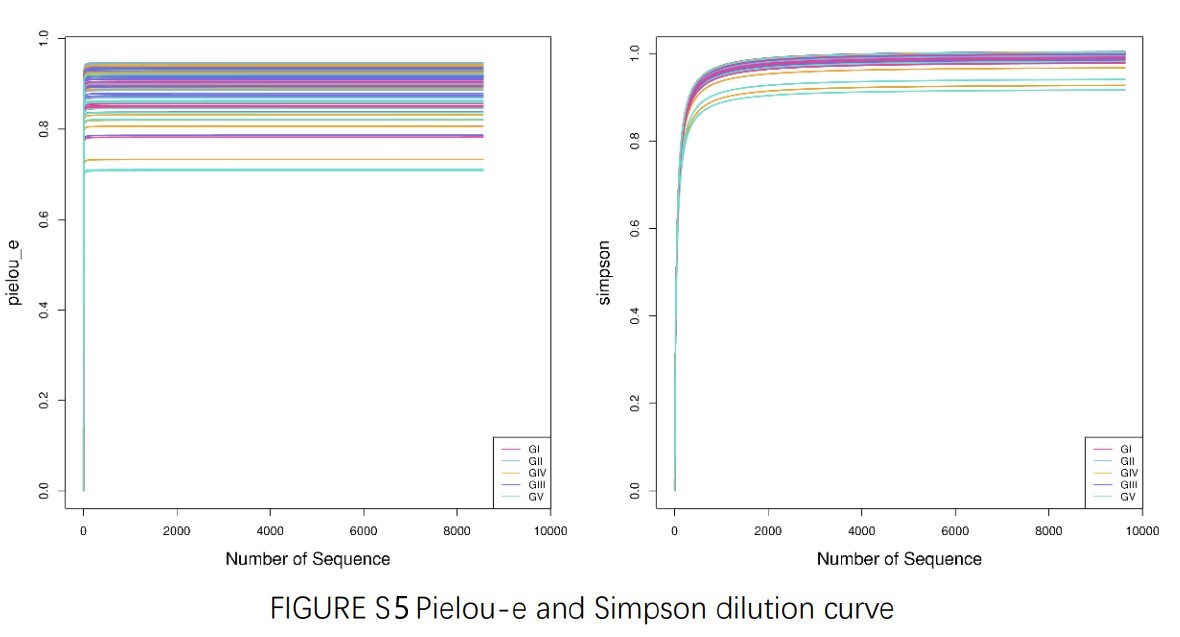

Supplement: Supplementary file 9 [file Image_5.JPEG]

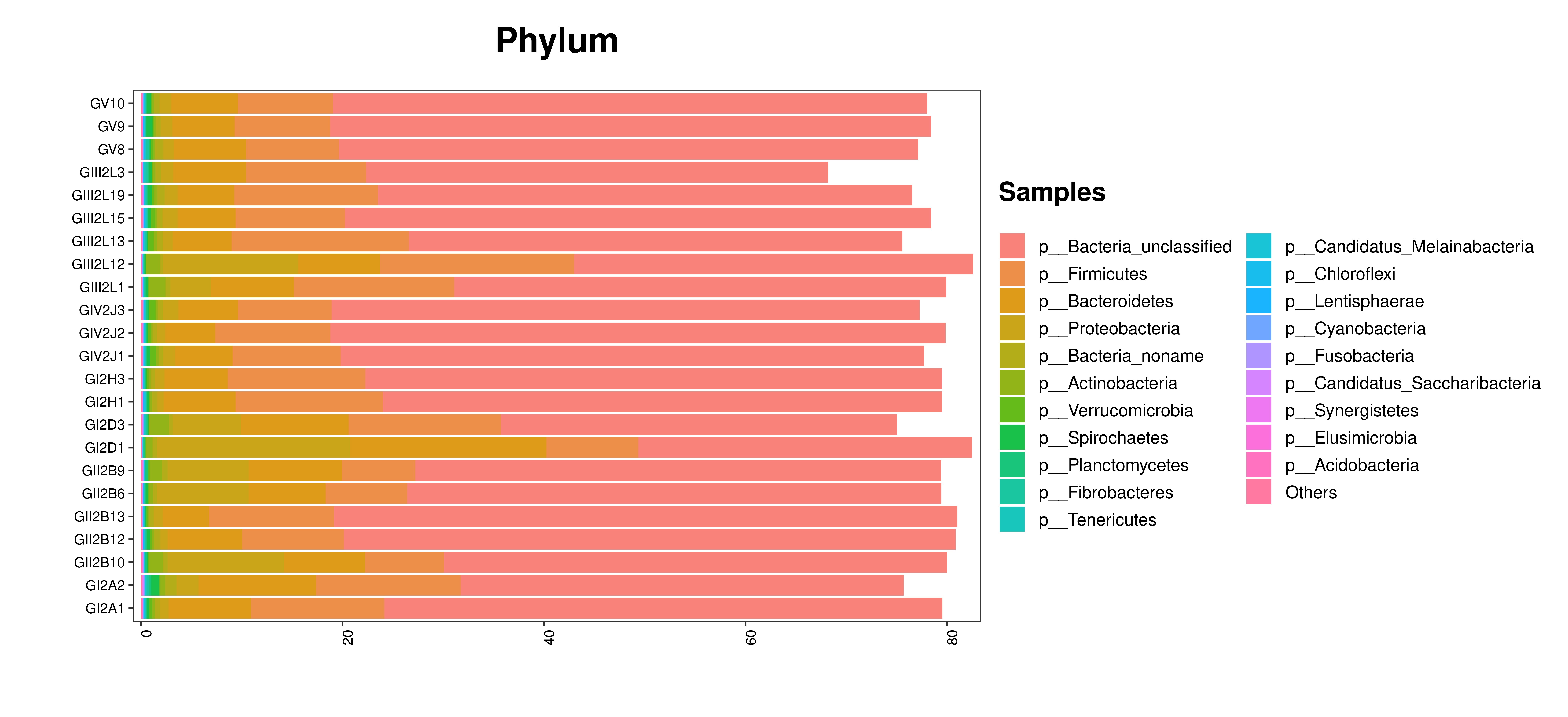

Supplement: Supplementary file 10 [file Image_6.JPEG]

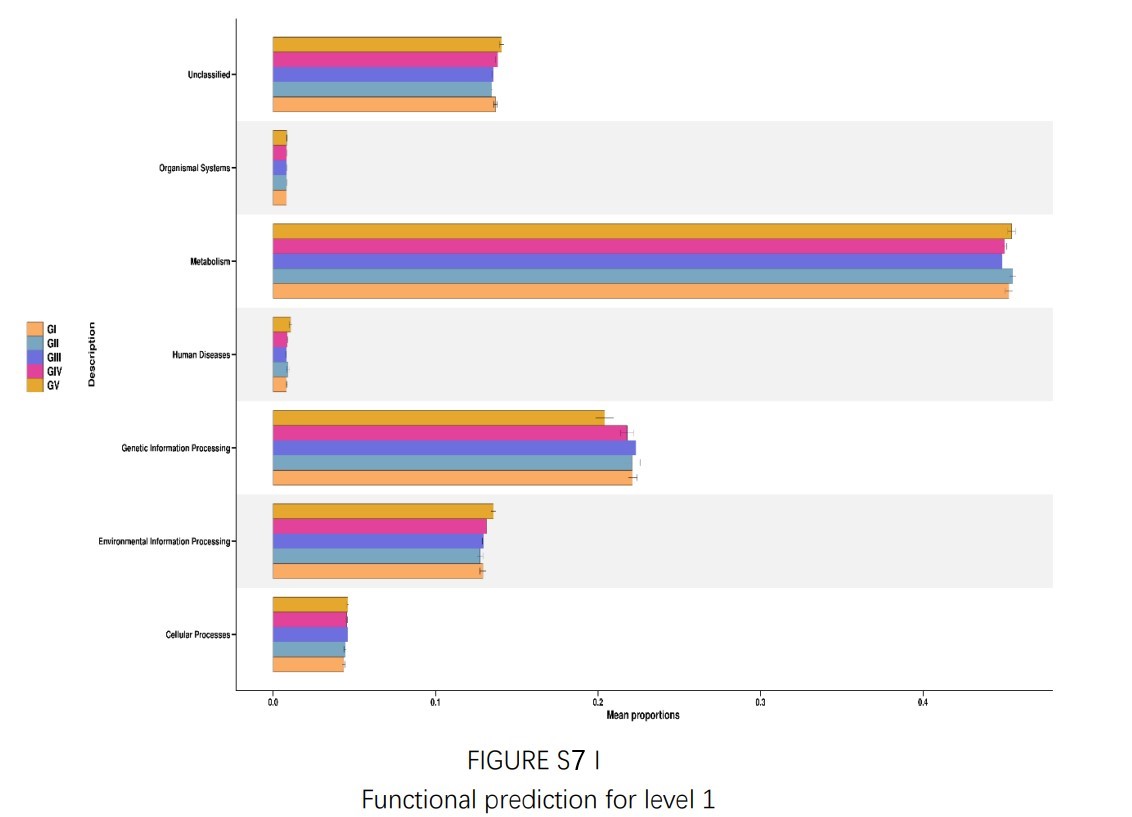

Supplement: Supplementary file 11 [file Image_7.JPEG]

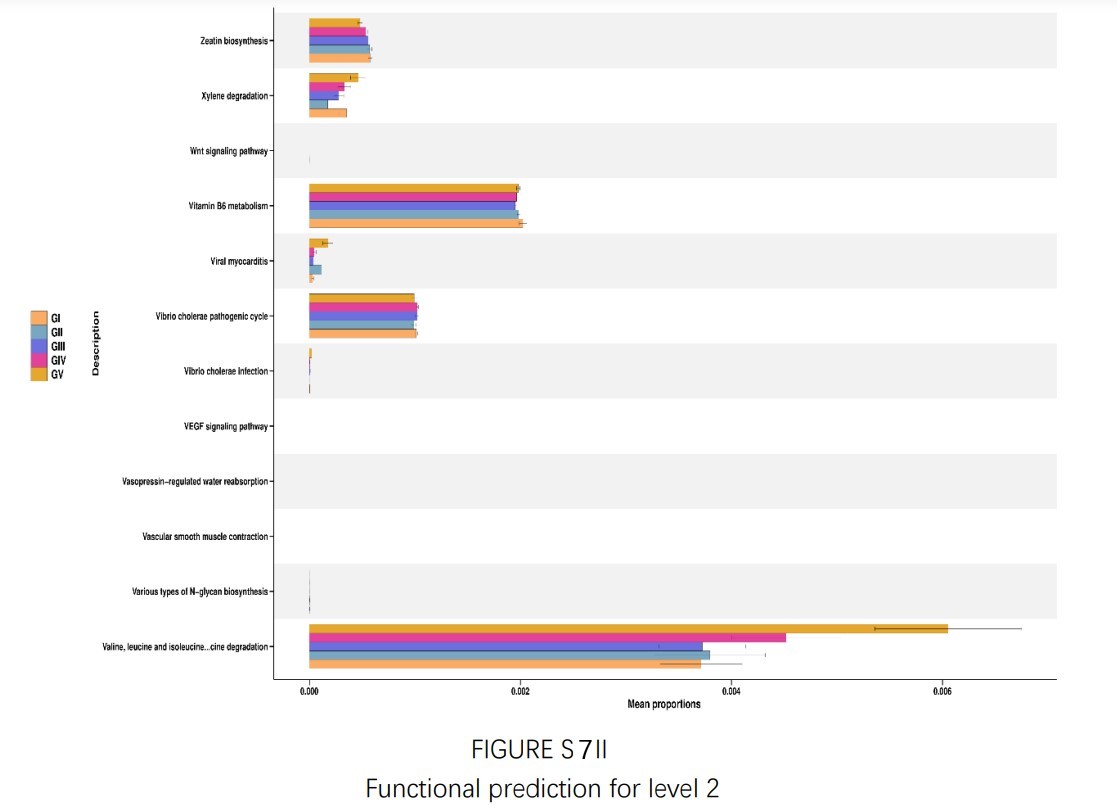

Supplement: Supplementary file 12 [file Image_8.JPEG]

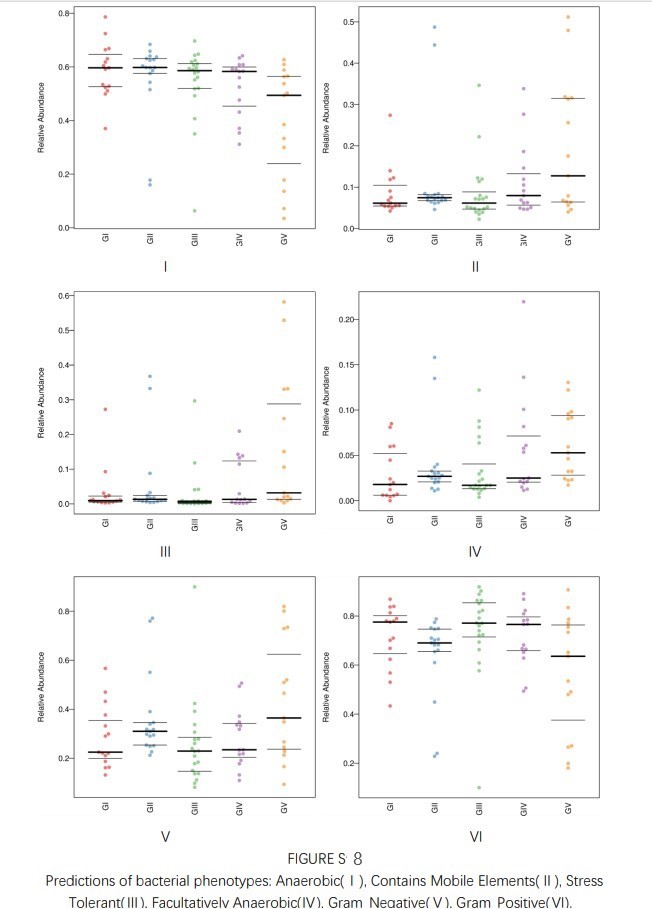

Supplement: Supplementary file 13 [file Image_9.JPEG]
